# Supplementary material for: A Global Screen for Assembly State Changes of the Mitotic Proteome by SEC-SWATH-MS
Source: Cell Syst. 2020 Feb 26;10(2):133–155.e6. doi: 10.1016/j.cels.2020.01.001 (PMC7042714; doi:10.1016/j.cels.2020.01.001)

Q03519 | TAP2_HUMAN | TAP2 ABCB3 PSF2 RING11 Y1
Monomer MW [kDa]: 75.664 Monomer expected elution fraction: 43

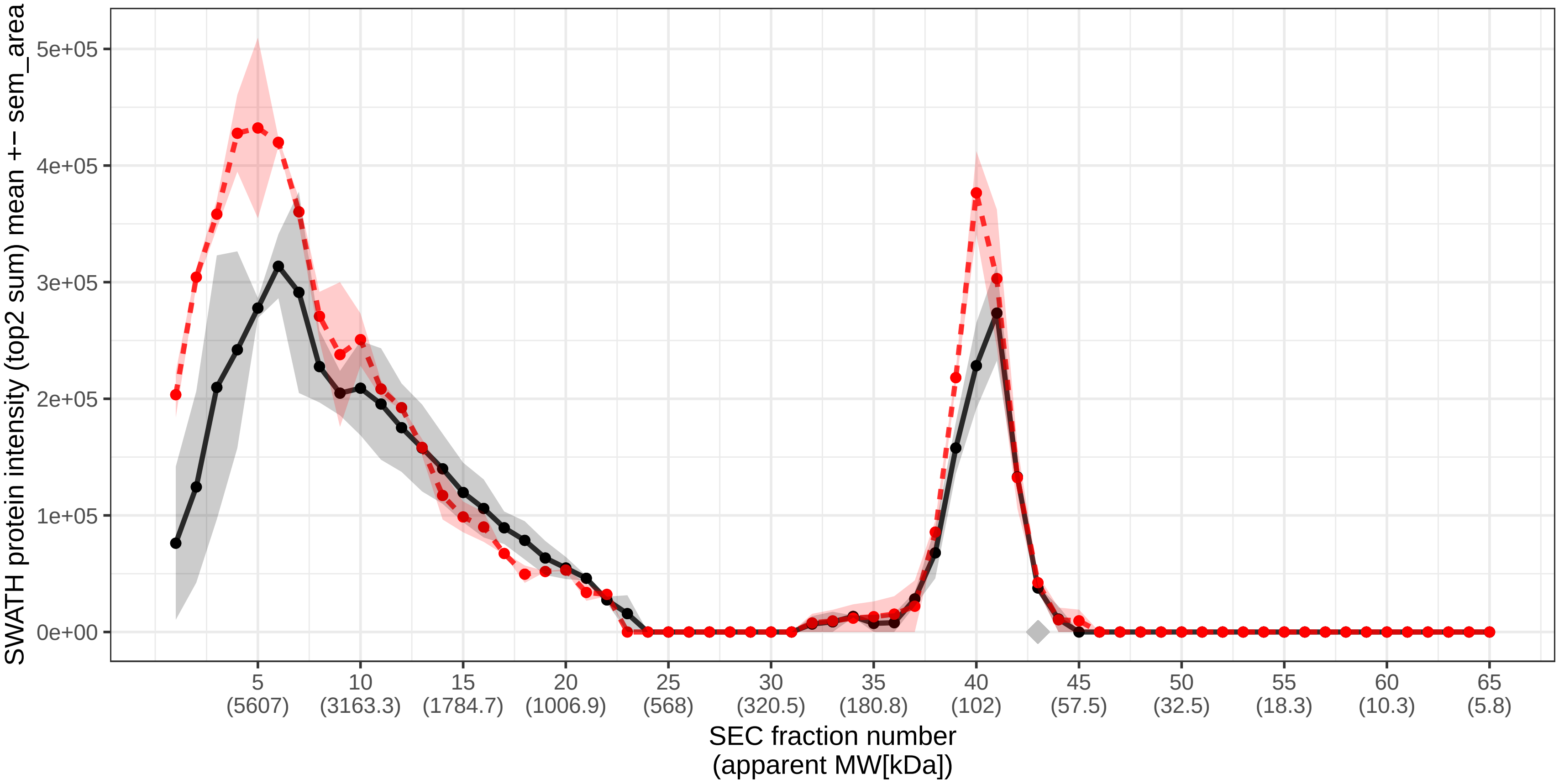

Supplement: Data S1. SEC-SWATH-MS Protein Chromatograms, Related to Figure 1 [file mmc6.zip › SECchrom_Q03519_TAP2_HUMAN_TAP2_ABCB3_PSF2_RING11_Y1.pdf]
